# Rho Kinase (ROCK) Inhibitors for the Treatment of Glaucoma

**DOI:** 10.2174/0113894501286195231220094646

**Published:** 2024-02-07

**Authors:** Junhui Wu, Jing Wei, Haoliang Chen, Yalong Dang, Fang Lei

**Affiliations:** 1 Department of Ophthalmology, the First Affiliated Hospital of Henan University of Science and Technology/College of Clinical Medicine of Henan University of Science and Technology, Luoyang, China;; 2 Department of Ophthalmology, Sanmenxia Eye Hospital/Sanmenxia Central Hospital Affiliated to Henan University of Science and Technology, Sanmenxia, China;; 3 Department of Ophthalmology, Henan University of Science and Technology, Luoyang, China

**Keywords:** Glaucoma, intraocular pressure, Rho kinase inhibitor, trabecular meshwork, neuroprotective, ophthalmology

## Abstract

Glaucoma is the most common cause of irreversible blindness worldwide. It is characterized by progressive optic nerve degeneration and loss of visual field. Pathological increased intraocular pressure is its main modifiable risk factor. Rho kinase inhibitors are developed as a new class of glaucoma medication that increases outflow facility from the conventional aqueous humor outflow pathway. Additionally, they also have neuroprotective and anti-scarring effects that can might increase the success rate of glaucoma filtration surgery. This review aims to summarize the current concept of Rho kinase inhibitors in the treatment of glaucoma from beach to bedside.

## 1 INTRODUCTION

Vision loss not only affects the quality of life of patients but increases psychological challenges, compromises educational attainment, and places a substantial burden on the economy [1]. Glaucoma is a leading cause of irreversible blindness worldwide [2]. The number of patients with glaucoma is estimated to exceed 100 million by 2040 [3]. Glaucoma remains asymptomatic until the occurrence of vision loss, and approximately 50% of patients are thought to have undiagnosed and untreated glaucoma [4, 5].

**Fig. (1):**
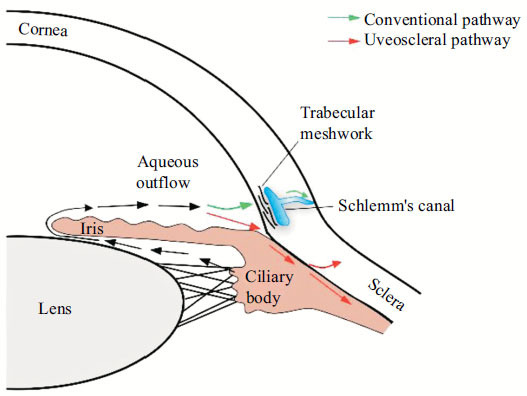
Aqueous humor outflow pathway. The green arrows indicate a conventional pathway. The red arrows indicate the uveoscleral pathway.

However, the pathogenesis of glaucoma remains poorly understood. Intraocular pressure(IOP) [6], age [7], genetics [8], excessive microglial activation [9], glutamate toxicity [10] and nitric oxide synthase insufficiency [11] have been implicated in the development of glaucoma. Among known risk factors, IOP is a substantial risk factor for the diagnosis and management of glaucomatous disease [6]. Moreover, IOP is the only quantitatively controllable parameter for glaucoma therapy [12, 13]. Typically, IOP depends on the balance between the production of aqueous humor (AH) in the unpigmented ciliary epithelium and the ciliary process and outflow of AH [14, 15]. There are two AH outflow pathways in the eye: trabecular (conventional) and uveoscleral (unconventional) (Fig. **1**) [14]. AH production remains normal in most glaucoma types, whereas AH outflow is partially blocked by the trabecular meshwork (TM), resulting in elevated IOP [16, 17].

Importantly, a well-controlled IOP can hinder the progression of glaucoma [2]. IOP reduction can be achieved using topical medication, laser therapy, or surgery [18]. Typically, medication is the preferred treatment choice for patients with glaucoma [18]. In recent decades, several small-molecule agents, such as cholinergic agents [19, 20], beta-adrenergic agonists [21, 22], alpha-adrenergic agonists [23], beta-blockers [24, 25], carbonic anhydrase inhibitors [26], prostaglandin analogs [27-29], and Rho-associated coiled-coil protein kinase (ROCK) inhibitors [30], have been developed and used to treat glaucoma. These molecular agents can lower IOP by reducing the rate of AH formation, increasing the outflow of AH from the unconventional outflow pathway, or decreasing the outflow resistance of AH in the conventional outflow pathway [23].

ROCK inhibitors reportedly regulate the remodeling of the TM cytoskeleton, increase AH outflow, and reduce IOP by inhibiting the Rho/ROCK signal pathway [31]. Thus, ROCK inhibitors are promising drug targets for the prevention and management of glaucoma [32]. This article provides a comprehensive and critical review of the current literature on ROCK inhibitors for glaucoma treatment.

## 2 RHO/ROCK PATHWAY

**Fig. (2):**
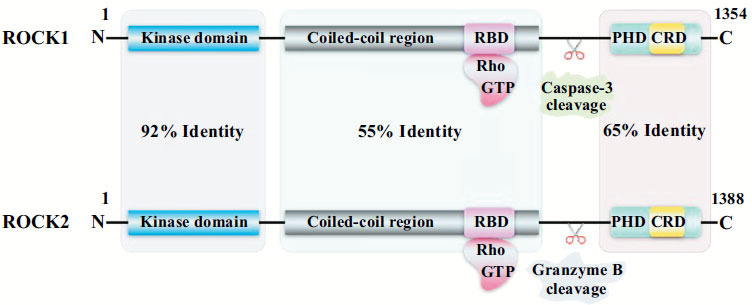
Schematic of Rho kinase. ROCK protein comprises three main domains: The kinase domain at the N-terminus; the coiled-coil-forming region with a Rho-binding domain (RBD), where RBD is a region that binds to RhoGTP and activates the ROCK protein; C-terminus, with a pleckstrin homology domain (PHD) containing an internal cysteine-rich domain (CRD). The C-terminus of ROCK1 and ROCK2 can be cleaved by caspase-3 and Granzyme B, respectively, to activate ROCK. N, N-terminal; C, C-terminus; ROCK, Rho-associated coiled-coil protein kinase; RBD, Rho-binding domain; PHD, pleckstrin homology domain; CRD, cysteine-rich domain; Rho GTP, Rho guanosine triphosphate.

The Rho family is an integral part of the Ras superfamily of small-molecule guanosine triphosphatases (GTPases), comprising RhoA, RhoB, and RhoC [33]. RhoA plays a pivotal role in regulating myosin contractility; RhoB regulates cytokine transport and cell proliferation; and RhoC plays a crucial role in cell motility [34-36]. Dysregulation of the Rho protein family plays a key role in the development of various human diseases. The Rho family is involved in fundamental biological processes in eukaryotic cells, including polarity, motility, morphogenesis, gene expression, cell division, and cytoskeletal reorganization [37, 38]. ROCK (ROCK1 comprises 1354 amino acids, and ROCK2 comprises 1388 amino acids) is a serine/threonine kinase and a major downstream effectors of Rho GTPases [34]. The kinase domain of ROCK is located at the N-terminus, followed by a coiled-coil-forming region with a Rho-binding domain (RBD), which contains a pleckstrin homology domain (PHD) with an internal cysteine-rich domain (CRD) that reduces the kinase activity *via* intramolecular interactions [39]. ROCK1 and ROCK2 exhibit an amino acid sequence homology of 65% [40], along with a kinase domain homology of 92%, indicating that the two subtypes have identical targets but distinct functions [34, 41] (Fig. **2**). Moreover, the two ROCK proteins have been identified in different tissues. ROCK1 is mainly found in the liver, lungs, kidneys, spleen, testes, and circulating inflammatory cells and is possibly associated with the formation of stress fibers. ROCK2 is mainly located in the heart, muscles (including smooth muscle), and brain and may be associated with cytoskeletal rearrangement, cell motility, or cell contraction [31, 40, 42].

**Fig. (3):**
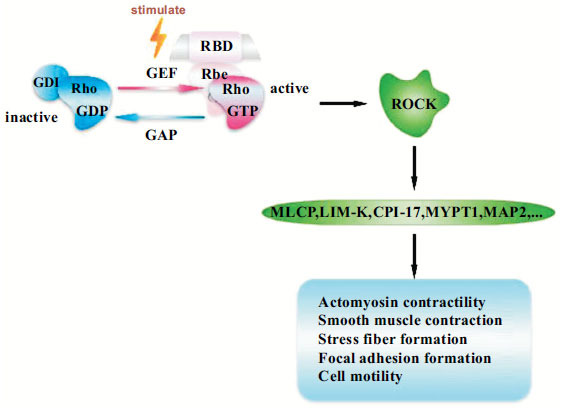
Schematic of the regulation of Rho/ROCK pathway function. ROCK can be activated by the exchange of GDP for GTP, which induces the phosphorylation of several substrates and plays a vital role in cell biological functions. GEFs, guanine nucleotide exchange factors; GAPs, GTPase activating proteins; GDIs, guanine nucleotide dissociation inhibitors; Rho GDP, Rho guanosine diphosphate; Rho GTP, Rho guanosine triphosphate; ROCK, Rho-associated coiled-coil protein kinase; LIM-K, LIM-kinase; MYPT1, myosin phosphatase target subunit 1; MAP2, microtubule-associated protein 2.

Rho is a molecular switch between the active (GTP-bound) and inactive (GDP-bound) conformations, which can control various cellular processes [43]. Guanine nucleotide exchange factors (GEFs) catalyze the sequential release and binding of guanine nucleotides, activating the function of guanine-nucleotide-binding proteins (G-proteins) by exchanging guanosine diphosphate (GDP) for guanosine triphosphate (GTP). GTPase-activating proteins (GAPs) promote the activation of G-proteins by stimulating GTP hydrolysis, whereas guanine nucleotide dissociation inhibitors (GDIs) suppress the spontaneous activation of G-proteins by extracting the inactive GTPase from the membranes, whose role appears to be to block spontaneous activation [37]. The Rho family GTPases are reportedly activated by several molecules such as endothelin-1 (ET-1), lysophosphatidic acid, thrombin, angiotensin II, cytokines, and transforming growth factor-β or *via* integrin activation after binding with the extracellular matrix (ECM) [32, 44]. Once in a GTP-bound state, Rho can activate numerous downstream effectors, including ROCK, which, in turn, induces the phosphorylation of several substrates, including myosin light chain phosphatase (MLCP), LIM-kinase (LIM-K), CPI-17, myosin phosphatase target subunit 1 and microtubule-associated protein 2, resulting in the regulation of actin cytoskeletal dynamics, actin-myosin contraction, cell adhesion, cell stiffness, cell morphology, cell proliferation, cell apoptosis and ECM reorganization [45] (Fig. **3**). ROCK has been explored as a potential target in various diseases, including glaucoma [34, 45-47].

## 3 ROCK INHIBITORS AND AH OUTFLOW

Approximately 80% of the AH flows out through the conventional pathway [14]. In addition, RhoA and ROCKs are expressed in the TM, juxtacanalicular tissue (JCT), Schlemm's canal (SC), and ciliary muscles (CM) of the outflow pathway [48-50]. Based on *in vitro*, *ex-vivo*, and *in vivo* evidence, ROCK inhibitors decrease IOP by inhibiting the Rho/ROCK signaling pathway to regulate cell contractility, SC permeability, ECM recombination, and phagocytosis in the conventional outflow pathway [30, 48, 49, 51-56] (Tables **1** and **2**).

### 3.1 ROCK Inhibitors and Preclinical Studies

#### 3.1.1 ROCK Inhibitors and the TM

The cytoskeleton is a three-dimensional network structure composed of protein fibers intertwined in the cell, which is composed of microtubules, microfilaments (specifically actin filaments) and intermediate filaments [57]. The cytoskeleton is highly organized in TM cells. TM has smooth muscle-like features [58]. Studies have shown that ROCK inhibitors can directly affect the TM to reduce IOP [49, 51]. The primary mechanism of action of ROCK inhibitors is to reduce MLC phosphorylation levels by activating MLCP [52-54, 59-62], leading to TM cell relaxation and disassembly of actin stress fibers and focal adhesions [48, 49, 51-56], and altering the contractility of TM cells to increase AH outflow facilities, leading to lower IOP [48, 51, 59, 61, 63, 64]. ROCK inhibitors affect TM cell contraction and regulate tissue contraction in the uveoscleral pathway [51, 64]. However, Nakajima E *et al.* studied that ROCK and its substrates are expressed higher in TM than CM, and Y-39983 exhibits dose-dependent relaxation of the carbachol-induced contraction state of monkey TM but has little effect on CM relaxation [48]. These studies suggest that ROCK inhibitors reduce IOP primarily through the TM pathway.

TM cells can engulf particulate matter and waste [65-69]. The phagocytosis of TM might be part of an essential self-cleaning mechanism [68]. Recently reported ROCK inhibitors may promote phagocytosis of TM cells to reduce IOP [70-72]. Yet further studies are still required.

Multiple studies of ROCK inhibitors in perfusion models and live animals have shown a reduction in IOP by increasing outflow facilities. Perfusion experiments conducted in live rabbits [30, 73-75], mice [54, 76, 77] and monkeys [78, 79], enucleated bovine [80], porcine [49, 70, 71], monkey eyes [81] and human eyes [82] have indicated ROCK inhibitors significantly increase the outflow facility of perfused eyes. However, the results of animal studies do not necessarily predict human outcomes. Therefore, clinical trials are needed to evaluate the efficacy and safety of ROCK inhibitors.

#### 3.1.2 ROCK Inhibitors and SC

Studies have shown that ROCK inhibitors increase AH outflow by increasing the permeability of Schlemm's canal endothelial  (SCE)-cells  [49, 83].  According  to Tanihara H *et al.*, Y-27632 could decrease ZO-1 and claudin-5 expression in the SCE cell monolayer, destroying tight junctions between cells and increasing paracellular permeability [84]. Treatment with ripasudil could increase the permeability of the SCE cell monolayer [54, 83]. However, Rao PV *et al.* reported that the endothelial lining was found to be intact after Y-27632 perfusion, as determined by transmission electron microscopy [49]. Therefore, it is necessary to further explore the role of ROCK inhibitors in lowering IOP.

#### 3.1.3 ROCK Inhibitors and the ECM

Accumulated evidence suggests that ROCK inhibitors may reduce the resistance to conventional AH outflow and lower IOP by inducing ECM reorganization and weakening cell binding to the ECM, resulting in more expansive empty spaces [85]. In addition, studies have shown that ROCK inhibitors could reverse the TGF-β2-induced ECM rearrangements [86-88]. However, the specific mechanism between the ECM and the Rho/ROCK pathway remains unclear, and whether ROCK inhibitors act on the ECM remains unresolved.

ROCK inhibitors reduce the IOP primarily *via* the conventional outflow pathway. In recent years, several review articles discussing the role of ROCK inhibitors in glaucoma have been reported [32, 45, 89-92].

### 3.2 Clinically Used ROCK Inhibitors

No new medications capable of impacting outflow resistance *via* the conventional pathway were introduced clinically until 2014, following which ripasudil was approved in Japan for the treatment of glaucoma and ocular hypertension (OHT). Other ROCK inhibitors have also been explored in clinical studies.

#### 3.2.1 Ripasudil (K-115)

Ripasudil (K-115)is an isoquinoline-sulfonamide derivative, with 50% inhibitory concentrations (IC50) of 0.051 and 0.019 μM against ROCK1 and ROCK2, respectively [30]. Ripasudil (Glanatec^®^ ophthalmic solution 0.4%, also known as K-115, Kowa Company, Ltd, Nagoya, Aichi, Japan) was approved in Japan for treating glaucoma and OHT in September 2014, at a dose of one drop administered twice daily. Ripasudil lowers IOP *via* direct action on the TM; increasing the conventional outflow pathway [93] (Fig. **4a**).

In a phase Iclinical trial in healthy volunteers [94], with single-instillation ripasudil (0.05%, 0.1%, 0.2%, 0.4%, and 0.8%), IOP decreased in a dose-dependent manner from baseline, with the maximum reduction reached after 2 hours. Instilling twice a day for seven days also reduced IOP. However, the ripasudil and placebo groups had no significant difference in IOP reduction. The sample size of their study was relatively small, and the findings may be limited. In the safety trial, it was found that with conjunctival hyperemia being the most common, more than half of the cases of mild to moderate severity, and most cases resolved spontaneously within 11/2 hours. It may be related to vasodilation caused by its relaxation of vascular smooth muscle [79].

In a randomized, multicentre, prospective, phase II study (JapicCTI-101015) [95], ripasudil (0.1%, 0.2%, and 0.4%) is used in patients with POAG or OHT twice a daily for eight weeks, a decrease in IOP could be found to be dose-dependent. In addition, a concentration of 0.4% ripasudil decreased to 4.5 mmHg after 2 hours of administration compared to the placebo. Moreover, after 8 hours of administration, there is still a reduction in IOP. Furthermore, in a randomized, open-label, placebo-controlled, multicentre, 24-hour time course study (JapicCTI-090708) [96], in POAG or OHT, the mean maximal IOP reduction of 0.4% ripasudil was -6.4 and -7.3 mmHg at 2 hours after the first and second treatment, respectively, and 0.2% and 0.4% of ripasudil was found to have a statistically significant reduction in IOP at 1 to 7 hours compared with placebo. The side effects of K-115 treatment in both clinical studies were mainly mild conjunctival hyperemia [95, 96]. In the phase II study, there was a dose dependency in the incidence of conjunctival hyperemia, and 0.1% to 0.4% of K-115 treatment was acceptably tolerated for 8 weeks [95]. Safety evaluation of K-115 for long-term treatment or in combination with other IOP-lowering eye drops is still needed. In the 24-hour time course study, no conjunctival hemorrhage was found [96].

A short-term (8-week) study of ripasudil-timolol and ripasudil-latanoprost, phase III study (JapicCTI-111700) found that after 2 hours of use, the ripasudil-timolol and ripasudil-latanoprost groups reduced -2.4 and -2.9 mmHg at trough levels and -2.2 and -3.2 mmHg at peak levels, respectively, compared with baseline mean IOP [97]. In the studies of ripasudil-timolol and ripasudil-latanoprost, the incidence of conjunctival hyperemia was 65.4% and 55.9%, respectively. Eye irritation, allergic conjunctivitis, and punctate keratitis were less common. Compared with the placebo, there was no significant decrease in trough levels in the ripasudil-latanoprost group in the primary efficacy endpoint. Another study showed that ripasudil had similar additive effects with prostaglandin analogs, β-blockers, and fixed combination drugs over a 52-week treatment period (JapicCTI-111565) [98]. In the study, more than 8 weeks after treatment with K-115, allergic blepharitis and conjunctivitis appeared, which were the main reasons for the discontinuation of ripasudil.

In a long-term (24 months), prospective, multicenter, open-label, phaseIVstudy [99-101] that investigated the long-term safety and effectiveness of ripasudil in over 3000 patients with POAG, normal tension glaucoma (NTG), secondary glaucoma (SG), ocular hypertension (OH) and primary angle-closure glaucoma (PACG), the reduction in IOP can be maintained for 24 months, except for patients with neovascular glaucoma, which may be related to pathogenesis [102] and subgroup size (n = 22). Furthermore, high baseline IOP affects the magnitude of IOP reduction after ripasudil treatment. Topical 0.4% ripasudil recipients with uveitic glaucoma and steroid-induced glaucoma were significantly larger in IOP-lowering effects than those of exfoliation glaucoma in a retrospective multicentre study in 332 patients with three secondary glaucoma subtypes, which was likely related to the higher baseline IOP levels of uveitic glaucoma and steroid-induced glaucoma [103]. In addition, ripasudil has a positive anti-inflammatory effect in patients with glaucoma secondary to uveitis, as inflammation-related scores are significantly reduced after treatment.

A study [104] of observation of ripasudil-induced long-term blepharitis found a higher cumulative incidence (34.6%) at 24 months with the use of ripasudil, which includes eyelid inflammation (erythema and edema), scaling of the skin, and pruritis, but did not involve conjunctival inflammation. It was found that blepharitis usually occurs on average nine months after treatment with ripasudil. After the demonstration of blepharitis, most patients discontinue the medication, which is the most common reason for withdrawal. Furthermore, another study found that patients had the highest risk of blepharitis after receiving ripasudil for 6 to 12 months [101]. Further research is needed to determine the exact mechanism by which adverse events of inflammation occur.

#### 3.2.2 Netarsudil (AR-13324)

Netarsudil (AR-13324) is one of a class of amino-isoquinoline amide ROCK inhibitors, a small molecule inhibitor of ROCK and norepinephrine transporters (NET). In December 2017, netarsudil (Rhopressa^®^ 0.02% ophthalmic solution, also known as AR-13324, Aerie Pharmaceuticals, Bedminster, NJ, US) was approved by the US FDA for the treatment of patients with open-angle glaucoma or ocular hypertension, at a dose of one drop once daily [105]. Netasudil may have one or more lower IOP mechanisms [increased AH outflow through the conventional trabecular outflow pathway [76, 82, 106], decreased AH production [106, 107], and reduced episcleral venous pressure (EVP) [107, 108]. It was proposed that this second mechanism of IOP lowering may be related to the Norepinephrine transporter (NET) inhibition, which can inhibit the reuptake of norepinephrine and may increase adrenergic signaling [107] (Fig. **4b**).

In an open-label, non-comparative, phaseIstudy (NCT01997879) in 18 healthy volunteers, topical 0.02% netarsudil administered once daily in the morning in each eye for 8 days afforded clinically and statistically significant reductions in IOP, which were more pronounced than those at baseline [109]. The most common adverse effect was conjunctival hyperemia, which was generally mild and short-lived.

In a double-blind, randomized, 28-day study comparing 0.01% and 0.02% netarsudil administered daily, the 0.02% formulation elicited a slightly more prominent IOP reduction than the 0.01% formulation, and both were statistically significant. In unmedicated patients with IOP ranging from 22 to 35 mmHg, netarsudil (0.02%) reduced IOP by ~1 mmHg when compared with latanoprost [110]. A double-masked, randomized, phaseIIstudy (NCT03233308) [108], administered 0.02% netarsudil once daily for 7 days in patients with POAG or OHT. The authors found that IOP reduction could be achieved by improving outflow, lowering EVP, and increasing trabecular outflow by approximately 35% and 25%, respectively, in the netarsudil group compared with baseline and control groups. In addition, treatment with netarsudil reduced the EVP ~10% from that at baseline. Clinical trial data shows that conjunctival hyperemia is generally mild and short-lived. In a study assessing a 0.02% netarsudil administration once a night, only 24% to 35% of patients presented with conjunctival hyperemia (mild and moderate) at morning visits on days 7 and 28 [110].

Two large, double-masked ROCKET-1 and ROCKET-2 (NCT02207491 and NCT02207621) phase III clinical trials involved patients with a baseline IOP maximum < 25 mmHg. Comparing once-daily 0.02% netarsudil with twice-daily 0.5% timolol revealed that neither once nor twice-daily netarsudil was inferior to timolol [111]. Furthermore, in another phase III study (NCT02558374), the IOP-lowering effects of netarsudil were non-inferiority to those of timolol in patients with a baseline IOP < 27 mm Hg and <30 mmHg [112]. In a retrospective study, treatment with netarsudil could substantially reduce IOP at 1, 3, 6, and 12 months when compared with those at baseline in patients with POAG and SG [113]. In addition to conjunctival hyperemia, another commonly observed ocular adverse event among netarsudil-treated patients was cornea verticillata (mild or moderate). Conjunctival hemorrhage (mild or moderate) and cornea verticillata are benign lipid microdeposits collected in the corneal epithelium. Patients who developed cornea verticillate did not experience a change in visual acuity. Following drug discontinuation, the symptoms typically disappeared within 13 weeks. Conjunctival hemorrhage is characterized by slight petechial bleeding [111, 112]. Other uncommon ocular adverse events associated with once-daily netarsudil administration include lacrimation, subconjunctival hemorrhage, blurred vision, instillation site pain, erythema, and erythema of the eyelid [110-112].

A fixed-dose combination (FDC) of netarsudil 0.02% with latanoprost 0.05% (Rocklatan^®^ PG324) was approved in the USA (March 2019) for the treatment of POAG and OHT [45]. The FDC outperformed both netarsudil and latanoprost alone in a double-masked, randomized, phase II clinical trial over 28 days [114], The FDC (with netarsudil at 0.02%) induced a -1.9 mmHg reduction in IOP when compared with latanoprost alone and a -2.6 mmHg reduction compared with netarsudil alone. In the double-masked, randomized, phase III clinical trials MERCURY-1 [115, 116] (12-month) and MERCURY-2 [117] (3 months), patients were randomized to once-daily netarsudil/latanoprost FDC, netarsudil, or latanoprost for 3 months or 12 months. In the MERCURY-2 (NCT02674854) study, treatment with the netarsudil/latanoprost FDC reduced the IOP more effectively than netarsudil or latanoprost alone, with IOP reduced by 2.2 to 3.3 mmHg and 1.5 to 2.4 mmHg, respectively. The American Academy of Ophthalmology considers a 20%-30% reduction in IOP a reasonable goal for first-line treatment in patients with open-angle glaucoma; this is consistent with the 3-month efficacy of another similarly designed MERCURY-1 study (NCT02558400), in which 42.1% of patients achieved a mean circadian IOP of 1≤5 mmHg with netarsudil/latanoprost FDC at month 3 when compared with 15.8% and 18.3% achieved with netarsudil and latanoprost monotherapy, respectively. Considering the accumulated data, netarsudil/latanoprost FDC could achieve a more substantial reduction in IOP to delay or prevent further visual field defects. Netarsudil/latanoprost FDC (Rocklatan^®^) combines the main mechanisms of action of the two drugs to lower IOP.

Recently, Q.K. Jiang *et al.* [118] developed an effective, safe, once-daily fixed-combination timolol-netarsudil-latanoprost ophthalmic solution (FC-TNL). FC-TNL potentially reduced dosing frequency to improve patient compliance and reduced ocular side effects. The stability and safety of FC-TNL have been documented both *in vitro* and *in vivo*. In animal experiments, once-daily FC-TNL continuously lowered IOP for 24h and delayed the death of retinal ganglion cells (RGCs) to protect vision. Although clinical investigations are pending, FC-TNL is an ideal preparation for treating glaucoma.

#### 3.2.3 Verosudil (AR-12286)

Verosudil (AR-12286), a water-soluble amino-isoquinoline amidesis ROCK inhibitors, is a potent, selective ROCK inhibitor [119],which was developed by Aerie Pharmaceuticals developed it53. AR-12286 reduces IOP by relaxing TM cells, increasing SCE cell permeability, and reducing abnormal accumulation of ECM [120].

In a single-center, crossover, phase I study of 18 regular adult volunteers, anministering 0.5% AR-12286 in the morning for 8 days resulted in a mean IOP reduction of up to 7 mmHg, which was clinically and statistically significant [119].

A double-blind, randomized phase II study (NCT00902200) of AR-12286 (0.05%, 0.1%, and 0.25%) in patients with POAG or OHT produced dose-dependent, statistically significant, and clinically significant reductions in the mean IOP [121]. Administration of AR-12286 (0.5 and 0.7%, respectively) for 24 weeks substantially reduced IOP in patients with exfoliative syndrome, OHT, and exfoliative glaucoma, indicating that the drug was well tolerated [122].

The most common side effect of AR-12286 is conjunctival hyperemia; other side effects include ocular irritation, increased lacrimation, and blurred vision [119-122]. Given that the IOP reduction elicited by AR-12286 is less than that achieved with natersudil, Aerie Pharmaceuticals Inc. abandoned the further development of AR-12286 for glaucoma treatment in 2017 (Fig. **4c**) [89].

#### 3.2.4 SNJ-1656 (Y-39983)

SNJ-1656 (Y-39983) is a selective ROCK inhibitor derived from Y-27632 and is more potent than Y-27632 [79]. SNJ-1656 was the first selective ROCK inhibitor tested clinically in the human eye [123]. Animal study have shown that the IOP-lowering effects of Y-39983 in rabbits and monkeys by increasing conventional outflow [79].

A phase I clinical study [123] demonstrated that SNJ-1656 at concentrations ranging from 0.003% to 0.1% (0.003%, 0.01%, 0.03%, 0.05%, and 0.1%) reduced IOP in a dose-dependent manner in 45 healthy volunteers, and maximal IOP reduction was observed from 2 to 4h when compared with the placebo. Within 24h, the IOP slowly recovered until it approached the baseline value.

In a randomized, double-blinded, multicentre, phase II clinical study, Y-39983 substantially reduced the mean IOP in patients with POAG or OHT [124]. The results of the phase 3 trial of SNJ-1656 for glaucoma or OHT have not yet been publicly announced.

During glaucoma treatment, the most common side effect associated with SNJ-1656 is mild conjunctival hyperemia, which spontaneously disappears after the discontinuation of medication use123. Other adverse event included punctate keratitis and mild hepatic dysfunction (Fig. **4d**) [124].

### 3.3 Other ROCK Inhibitors in Development

Fasudil (HA-1077) is mainly used to prevent and improve cerebral ischemia and vasospasm caused by subarachnoid hemorrhage surgery [125]. The IOP-lowering mechanism of HA1077 may be related to changes in TM cell behavior [74]. To date, only one clinical study has examined the application of HA1077 in glaucoma [126]. Four patients with unilateral end-stage POAG were treated with 0.5% fasudil (in three eyes) or 1.2% fasudil (in one eye) administered twice daily for eight weeks. Fasudil (0.5% and 1.2%) showed peak effects 2-4h after treatment and substantially reduced IOP. Treatment with 1.2% fasudil elicited the most substantial IOP reduction (up to -12mmHg).

Currently,multiple ROCK inhibitors are in development, including PHP-201 (AMA-0076) (NCT02136940, NCT01693315), ATS-907 (NCT01668524, NCT01520116), INS-117548 (NCT00767793), although clinical trials have been completed, data is yet to be published (Fig. **4e**) [92].

## 4 ROCK INHIBITORS AND NEUROPROTECTION

Over the past few years, the neuroprotective and axonal regeneration potential of ROCK inhibitors have been reported [127, 128]. RhoA protein levels in glaucoma optic nerve head (ONH) were substantially higher in patients with glaucoma than those in normal controls [50]. The RhoA/ROCK pathway plays a role in the pathophysiology of glaucoma-induced optic nerve damage [129]. The central role of ROCK inhibitors is to promote axon regeneration by increasing the optic nerve blood flow, which may help treat damaged optic neurons [130, 131]. Y-39983 was found to promote axonal regeneration of damaged RGCs [130, 132, 133] and increased blood flow in the ONH [130]. Furthermore, Y-39983 could downregulation active-RhoA, ROCK1, and ROCK2 expression, accompanied by the emergence of a large number of regenerating axons [133]. Moreover, fasudil was found to improve damaged ONH blood flow in rabbit models of ocular circulation impairment [131]. Y-27632 efficiently promoted the regeneration of RGC axons in a rat optic nerve crush model, whereas dimethylfasudil only showed a tendency to increase outgrowth *in vivo* [134]. In contrast, fasudil injection was ineffective in regenerating RGC axons in adult cats [135]. Topical administration of a netarsudil could promote RGC survival and regeneration after optic nerve injury [136]. Oral K-115 administration delayed RGC death after optic nerve crushing in mice and elicited a substantial protective effect [137]. Developing a more effective neuroprotective strategy will provide novel avenues for glaucoma therapy. The role of ROCK inhibitors in preventing RGC death has been observed in animal models of glaucoma. However, the neuroprotective effects of ROCK inhibitors have not been demonstrated in patients with glaucoma.

## 5 ROCK INHIBITORS AND CONJUNCTIVAL SCARRING AFTER GLAUCOMA SURGERY

In patients with glaucoma, filtration surgery is necessary when medications and laser treatments fail. Filtration surgery has been performed for decades, and postoperative scarring remains the leading cause of surgical failure [138]. Reportedly, ROCK inhibition may inhibit scarring [139]. During surgery, antimetabolites such as 5-fluorouracil (5-FU) and mitomycin C (MMC) are used to reduce postoperative scarring during wound healing [140, 141]. However, the use of these agents may be accompanied by other complications [142]. Therefore, the development of novel agents that control postoperative scar tissue formation without side effects would be valuable. TGF-β is closely related to fibroblast proliferation, and ROCK inhibitors block subsequent TGF-β-induced myofibroblast transdifferentiation [142, 143]. Based on *in vitro* studies, AMA0526, Y-27632, and H-1152P suppress the expansion, adhesion, and contraction of human tenon fibroblasts [139, 144, 145]. Additionally, AMA0526 and Y-27632 were shown to effectively prevent fibroproliferation and scar formation in a rabbit glaucoma surgery [139, 146]. Accordingly, ROCK inhibitors may be an effective anti-scarring agent after glaucoma filtering surgery. Therefore, the use of ROCK inhibitors in patients who have undergone filtration surgery may reduce the IOP and limit scarring. However, further preclinical and clinical studies are required to confirm this effect.

## CONCLUSION

In conclusion, ROCK inhibitors decrease IOP by inhibiting the Rho/ROCK signaling pathway to regulate cell contractility, SC permeability, and ECM recombination in the conventional outflow pathway, and may provide neuroprotection, and prevent scarring after glaucoma filtering surgery. In patients with glaucoma and OHT, ROCK inhibitors could be employed as second-line or adjunct agents with IOP-lowering drugs possessing alternate mechanisms to promote adherence to long-term treatment. ROCK inhibitors induce relaxation of vascular smooth muscle explaining the high incidence of conjunctival hyperemia. Other commonly observed ocular adverse events included conjunctival hemorrhage, cornea verticillate, blepharitis and allergic conjunctivitis. Although some patients withdrew from clinical trials due to adverse events, the side effects of ROCK inhibitors were mild and moderate, and symptoms could disappear after discontinuation. ROCK inhibitors provide clinicians with additional IOP reduction, potentially preventing visual field loss and disability. The development of ROCK inhibitors that maximize drug penetration and minimize local adverse effects is imperative; for example, they can be used for vehicle embedding or forming sustained-release agents

## Figures and Tables

**Fig. (4a):**
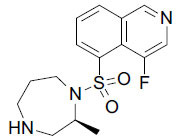
Chemical structure of ripasudil.

**Fig. (4b):**
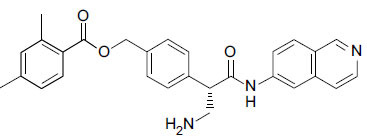
Chemical structure of netarsudil.

**Fig. (4c):**
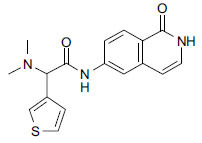
Chemical structure of AR-12286.

**Fig. (4d):**
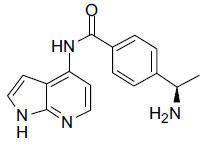
Chemical structure of Y-39983.

**Fig. (4e):**
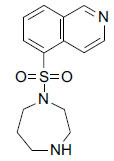
Chemical structure of fasudil.

**Table 1:** Effect of ROCK inhibitors on aqueous humor outflow *in vitro* and *ex-vivo*.

**Model**	**Effect**	**References**
Cultured human trabecular meshwork cells	Y-27632 destroyed stress fibers and impaired adhesion formation. Y-27632 caused reversible changes in cell shape and decreased staining for actin, focal adhesions, and protein phosphotyrosine. K-115 significantly inhibited TGFβ2-induced stress fiber formation, smooth muscle actin expression, and phosphorylation of both myosin light chain and cofilin. AR-13324 disrupted focal adhesions. H-1152 treats human trabecular meshwork cells with significantly reduced basal levels of MLC phosphorylation. HA1077 disrupted F-actin bundles and impaired focal adhesion formation. SB772077B leads to changes in cell shape and a decrease in actin stress fibers and extracellular matrix proteins. AMA0076 led to a decrease in actin bundles and focal adhesions.	[51] [49] [54] [53] [62] [74] [55] [56]
Cultured monkey trabecular meshwork cells	K-115 caused trabecular meshwork cells morphological changes and destruction of actin bundles.	[83]
Cultured porcine trabecular meshwork cells	K-115 can reduce trabecular meshwork stress fibers and increase phagocytosis and migration. AR-13324 disrupted actin stress fibers in primary porcine trabecular meshwork cells.	[70] [53]
Cultured human Schlemm’s canal cells	Y-27632(10 mM) increased the permeability of Schlemm’s canal cell monolayer (80%) and decreased myosin light chain phosphorylation. Y-27632 increased the permeability in Schlemm’s canal cells.	[49] [54]
Cultured monkey Schlemm’s canal cells	K-115 significantly decreased transendothelial electrical resistance, increased the transendothelial flux of fluorescein isothiocyanate-dextran, and disrupted cellular localization of ZO-1 expression in Schlemm’s canal cell monolayers.	[83]
Bovine trabecular meshwork strips	Y-27632 inhibits carbachol or endothelin-1-induced contraction of bovine trabecular meshwork strips.	[61]
Bovine ciliary muscle strips	Y-27632 led to the relaxation of the carbachol-precontracted ciliary muscle strips in a dose-dependent manner. HA1077 led to the relaxation of the smooth muscle in a dose-dependent manner.	[51] [74]
Rabbit ciliary muscle	Y-27632 inhibited the carbachol-induced constriction of rabbit ciliary muscle.	[64]
Enucleated human eyes	AR-13324 acutely increased the outflow facility by expanding the juxtacanalicular connective tissue and dilating the episcleral veins.	[82]
Enucleated porcine eyes	Y-27632 (10-100 mM) Increased aqueous humor outflow facilities for nucleated pig eyes (40%-80%). H-1152 causes a dose-dependent increase in the outflow facility of enucleated porcine eyes. RKI-1447 significantly reduces intraocular pressure by destroying trabecular mesh stress fibers and increasing trabecular mesh phagocytosis	[49] [62] [71]
Enucleated bovine eyes	Y-27632 increases the physical separation between the parallel connective tissue and the inner wall.	[80]
Enucleated monkey eyes	Y-27632 increases the outflow facility by redistributing aqueous outflow through a larger area in the juxtacanalicular connective tissue.	[81]

**Table 2:** Effect of ROCK inhibitors on aqueous humor outflow channels *in vivo*.

**Model**	**Effect**	**References**
Living rabbit eyes	Y-27632, when administered topically, intracamerally, or intravitreally, reduced intraocular pressure and increased the outflow facility. Y-27632 reduced intraocular pressure in rabbits by topical instillation. K-115 demonstrated a significant intraocular pressure-lowering effect in rabbits. K-115 lowered intraocular pressure by increasing conventional outflow facilities and has good visual penetration properties. AR-13324 had a decrease in intraocular pressure that lasted at least 24 hours after once-daily administration. AR-13324 lowered the episcleral venous pressure. Y-39983 intraocular pressure decreased most between 2 and 3 hours after treatment. H-1152P could observe intraocular pressure-lowering effects in normal rabbits and model rabbits with ocular hypertension. HA-1077 caused a time- and dose-dependent decrease in intraocular pressure in a rabbit eye hypertension model caused by water load. HA1077 induced a significant decrease in intraocular pressure in rabbits. AMA0076 effectively reduced intraocular pressure in ocular normotensive and acute hypertensive rabbits without causing distinct hyperemia.	[51] [64] [83] [30] [53] [107] [79] [75] [73] [74] [56]
Living mouse eyes	AR-13324 lowered intraocular pressure and increased outflow facility in mice. AR-13324 acted directly on regular outflow cells to reduce intraocular pressure.	[76] [77]
Living monkey eyes	Y-27632 dose-dependently increased outflow facility in living monkeys. K-115 had a significant intraocular hypotensive effect on monkeys. K-115 has significant ocular hypotensive effects *via* increases in conventional Outflow and has good ocular penetration characteristics in monkeys. AR-13324 produced significant reductions in intraocular pressure in monkeys that were sustained for at least 24 hours after once-daily dosing. AR-13324 could reduce intraocular pressure in monkey eyes by increasing aqueous humor outflow facilities and reducing aqueous humor outflow. Y-39983 showed a significant IOP-lowering effect within 2 to 7 hours after treatment, compared with vehicle-treated eyes.	[78] [83] [30] [53] [106] [79]

## LIST OF ABBREVIATIONS

CM: Ciliary Muscles
CRD: Cysteine-Rich Domain
ECM: Extracellular Matrix
EVP: Episcleral Venous Pressure
FDC: Fixed-Dose Combination
GDP: Guanosine Diphosphate
GTP: Guanosine Triphosphate
IOP: Intraocular Pressure
JCT: Juxtacanalicular Tissue
NET: Norepinephrine Transporters
SC: Schlemm's Canal
